# Unraveling Residual Stress Distribution Characteristics of 6061-T6 Aluminum Alloy Induced by Laser Shock Peening

**DOI:** 10.3390/ma17143484

**Published:** 2024-07-14

**Authors:** Qian Wang, Yaqiong Ge, Jingjia Chen, Tosei Suzuki, Yoshihiro Sagisaka, Ninshu Ma

**Affiliations:** 1 Joining and Welding Research Institute, Osaka University, Osaka 567-0047, Japan; 2 College of Materials Science and Engineering, Taiyuan University of Science and Technology, 66 Waliu Road, Taiyuan 030024, China; 3 Hamamatsu Technical Support Center, Industrial Research Institute of Shizuoka Prefecture, Shizuoka 431-2103, Japan

**Keywords:** laser shock peening, plasticity, residual stress, scanning path, modeling, aluminum alloy

## Abstract

Laser shock peening (LSP) is a powerful technique for improving the fatigue performance of metallic components by customizing compressive residual stresses in the desired near-surface regions. In this study, the residual stress distribution characteristics of 6061-T6 aluminum alloy induced by LSP were identified by the X-ray diffraction method, and their dependent factors (i.e., LSP coverage, LSP energy, and scanning path) were evaluated quantitatively by numerical simulations, exploring the formation mechanism of LSP residual stresses and the key role factor of the distribution characteristics. The results show that LSP is capable of creating anisotropic compressive residual stresses on the specimen surface without visible deformation. Compressive residual stresses are positively correlated with LSP coverage. The greater the coverage, the higher the residual stress, but the longer the scanning time required. Raising LSP energy contributes to compressive residual stresses, but excessive energy may lead to a reduction in the surface compressive residual stress. More importantly, the anisotropy of residual stresses was thoroughly explored, identifying the scanning path as the key to causing the anisotropy. The present work provides scientific guidance for efficiently tailoring LSP-induced compressive residual stresses to improve component fatigue life.

## 1. Introduction

Laser shock peening (LSP) is a surface treatment technique that utilizes high-energy laser pulses to generate shock waves on the surface of components, causing plastic deformation and compressive residual stresses to improve mechanical properties [1]. It was developed in the 1960s with the rise of pulsed lasers. Compared to conventional shot peening, LSP delivers deeper compressive residual stress [2,3,4,5], as well as the advantages of precise control of process parameters [6], no surface contamination [7,8], high processing efficiency [9], and environmental friendliness [9,10], which have led to its applications in the aerospace, automotive, medical, and energy sectors. In aerospace, LSP is highly popular for prolonging the fatigue life of critical components such as turbine engines and aircraft structures [1,2,11,12]. The ZAL Center for Applied Aeronautical Research in Hamburg has set up a €2.6 million LSP facility dedicated to the surface strengthening of critical aerospace components. Moreover, LSP on automotive engines and transmission components can improve fatigue life and corrosion resistance, making vehicles more efficient, reliable, and long-lasting [2,13]. Medical implants are surface treated via LSP to improve not only mechanical properties by inducing compressive residual stresses [14] but also biocompatibility by creating surface “anchorages” for cell integrins [15,16]. Its use in power generation and transmission components can help reduce maintenance costs, minimize downtime, and improve the efficiency and reliability of power generation systems [9,17].

Despite the various benefits, the widespread applications of LSP still face challenges and limitations. One major challenge is the high cost of equipment and maintenance, but advancements in laser technology are expected to alleviate this issue [18,19]. Currently, the lack of standardization of process parameters poses a pressing concern. Residual stress [2,20,21], surface topography [22,23], and fatigue life [24,25] are valuable indicators to evaluate the LSP role. In particular, achieving an appropriate residual stress distribution is recognized as vital for improving the mechanical properties of metallic materials. A substantial body of experimental research has shown that LSP parameters, such as laser pulse energy, laser spot size, laser power intensity, laser pulse duration, and laser coverage, influence the generation of compressive residual stresses in various metallic materials [5,26,27,28]. Adjusting the process parameters can obtain the optimal residual stress distribution to maximize the mechanical properties. Nevertheless, it is difficult to accurately grasp the dynamic response process of the LSP target and its correlation with the final residual stress distribution only experimentally. Numerical simulation is an effective means to understand the relationship between LSP parameters and residual stresses [5,27,28,29,30,31]. Due to the complexity of shock wave propagation in metallic components, it is crucial to perform the simulations correctly using suitable computational methodologies. Modeling has been instrumental in exploring the dependence of uneven surface residual stress distribution on stress wave propagation [32]. A mathematical model has been derived for the variation of residual stresses with surface curvature by finite element (FE) analysis [33]. Most current LSP simulations focus on residual stresses caused by one or several laser pulses. A single laser pulse covers quite a small area, whereas, in reality, the LSP required for the entire surface is achieved by scanning the laser pulses along a given path [5,21,25,34,35,36]. Meanwhile, the scanning path plays a role in LSP residual stresses. Zhang et al. [37] presented a backward solution using equilibrium constraints to compute in-plane residual stresses and eigenstrains, without accounting for path effects. Adu-Gyamfi et al. [38] and Xu et al. [39] explored the influence of scanning patterns. It was found that whether the residual stress distribution is affected by the path depends on the laser coverage, but the underlying cause remains unclear.

The purpose of this study is to give a clear understanding of the characteristics of LSP plastic deformation and residual stress distribution. Moreover, an advanced 3D nonlinear FE model was developed to reproduce the residual stress distribution at one point and one pass and to map laser pulses. The formation mechanism of LSP residual stresses was revealed. The influences of individual dependent factors, i.e., LSP coverage, LSP energy, and the scanning path, were discussed in detail to identify the key role factor of the distribution characteristics and elucidate the relationship between LSP parameters and final residual stresses. It is expected to provide scientific insights for LSP to tailor residual stresses efficiently.

## 2. Materials and Methods

### 2.1. Laser Shock Peening

**Figure 1:**
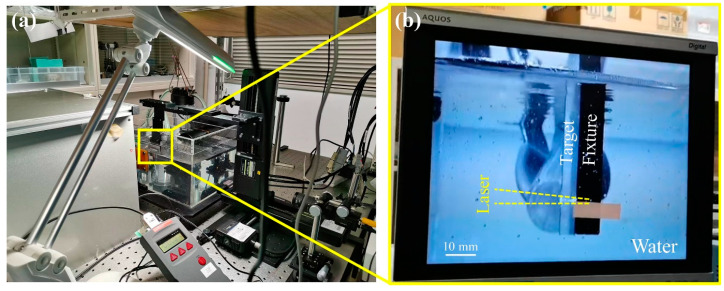
Photographs of the (**a**) laser shock peening set-up and (**b**) localized magnification underwater.

With a high strength-to-weight ratio, favorable corrosion resistance, and excellent machinability, 6061-T6 aluminum alloy (AA6061-T6) is extensively used in the aerospace and automotive industries. Hence, AA6061-T6 was selected as the target material in this study and cut into 5 mm × 50 mm × 50 mm sheets for use. Figure 1a displays the LSP equipment photograph. The nanosecond laser wavelength is 532 nm, the pulse duration ($T_{L}$) is 8 ns (full width at half-maximum (FWHM)), the pulse frequency is 10 Hz, and the pulse energy is up to 500 mJ, as listed in Table 1. The variation in pulse energy with time in a laser system is approximately Gaussian. The laser wavelength, frequency, and energy were set and presented through LSP equipment. The AA6061-T6 sheets were fixed under water and scanned by focusing the laser beam with a lens having a focal length of 50 mm. A filter was added to prevent water contamination. The LSP process can be observed synchronously through the camera, as shown in Figure 1b. The laser pulse energy (E) was set to 200 mJ, and the laser spot diameter (d) was controlled at 1 mm. The laser power intensity depends on the laser pulse energy, pulse duration, and spot diameter and can be defined as $I_{0}=\frac{4E}{\pi d^{2}T_{L}}$ [36]. It was determined to be 3.18 GW·cm^−2^. The peak pressure can be estimated by $P_{max}\left(GPa\right)=0.01\sqrt{\frac{\alpha }{2\alpha +3}}\sqrt{Z\left(g\cdot {cm}^{-2}\cdot s^{-1}\right)}\sqrt{I_{0}\left(GW\cdot {cm}^{-2}\right)}$ [40]; see Section 3.1 for more details. Zigzag scanning was performed at a scanning speed of 3 mm/s with intervals of 0.3 mm and 5 mm/s with intervals of 0.5 mm, respectively. Both LSP treatments had an area of 30 mm × 30 mm without a sacrificial layer. Coverage was estimated at 870% and 310%, respectively.

### 2.2. Surface Topography and Residual Stress Measurement

**Figure 2:**
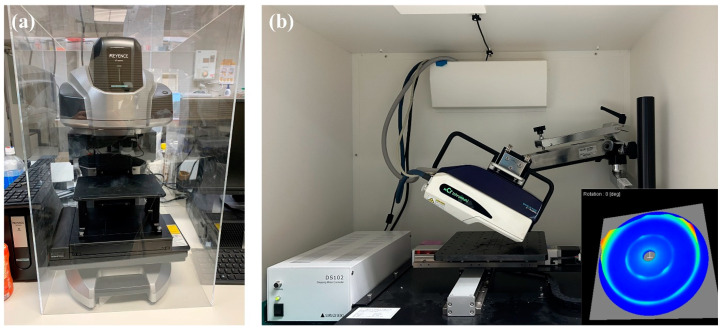
Measurement devices used for (**a**) surface topographies and (**b**) residual stresses.

Energy concentration is an intrinsic characteristic of lasers. LSP involves the usage of an extremely high-power laser (∼1013 W·m^−2^) to rapidly irradiate the surface of a water-covered specimen with nanosecond pulses, and the resulting plasma rapidly expands and generates shock waves [41,42], which leads to localized large plastic deformation and pronounced compressive residual stresses of the specimen. Hence, it is essential to determine the surface topographies and residual stresses. The surface texture and overall deformation after LSP were characterized by a non-contact wide-area 3D measurement system (VR-5000, KEYENCE Corp., Osaka, Japan), as shown in Figure 2a. The working distance is 75 mm, and the display resolution of height measurement is 0.1 μm.

Residual stresses were measured by the X-ray diffraction (XRD) method. Specifically, the XRD (cosα) method [43], as a non-destructive surface residual stress measurement method, was adopted using a portable X-ray residual stress analyzer (μ-X360s, Pulstec Industrial Co., Ltd., Hamamatsu-City, Japan), as shown in Figure 2b. Different from the XRD (sin2ψ) method, the XRD (cosα) method enables simultaneous acquisition of the entire Debye-Scherrer ring via a 2D sensor, making residual stress measurement more convenient and faster while ensuring the equivalent measurement accuracy. The collimator size was 1 mm. First, the sample was placed, and the X-ray tube of Cr was adopted with an incidence angle of 25° and a distance of 39 mm. Second, the point to be measured was irradiated for 30 s to obtain the residual stress. An automatic moving stage was utilized to measure the whole surface with 2 mm intervals automatically. The penetration depth of an X-ray is approximately 3–4 μm. The X-ray tube current and voltage are 1.50 mA and 30.00 kV, respectively.

## 3. Numerical Framework

### 3.1. Shock Wave Modeling

**Figure 3:**
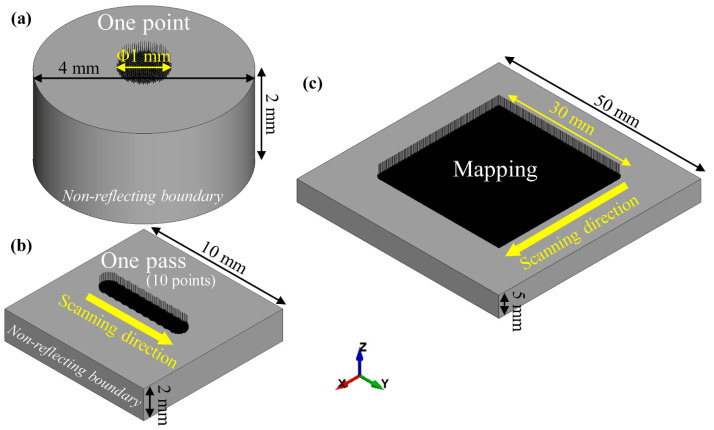
FE models built for laser shock peening AA6061-T6 simulations: (**a**) one point; (**b**) one pass; (**c**) mapping.

To better understand the LSP residual stresses and their formation mechanism, 3D nonlinear FE models of LSP from one point to one pass and mapping were established, as shown in Figure 3. Based on the experiments, all laser spot diameters were set to 1 mm. Generating a shock wave during LSP is equivalent to applying a dynamic pressure load to the target material, and the peak pressure has been evidenced to be estimated by the following equation: $P_{max}\left(GPa\right)=0.01\sqrt{\frac{\alpha }{2\alpha +3}}\sqrt{Z\left(g\cdot {cm}^{-2}\cdot s^{-1}\right)}\sqrt{I_{0}\left(GW\cdot {cm}^{-2}\right)}$, where α denotes the proportion of internal energy converted to thermal energy (a = 0.2, here), and Z refers to the reduced acoustic impedance between water and the target material AA6061-T6, expressed as $\frac{2}{Z}=\frac{1}{Z_{water}}+\frac{1}{Z_{target}}$, where $Z_{water}$ and $Z_{target}$ are the acoustic impedance of water and the target ($Z_{water}$ = 0.165 × 106 g·cm^−2^·s^−1^ and $Z_{target}$ = 1.5 × 106 g·cm^−2^·s^−1^, here), respectively. Accordingly, $P_{max}$ in this study was determined to be 2.3 GPa, with the pressure load applied in the form of a triangular pulse [44]. Hexahedral elements were used, with a size of ~0.05 mm. The target material was assumed to be a cylinder with a radius and height of 2 mm for one-point modeling (Figure 3a) and a cuboid with a thickness of 10 mm and a height of 2 mm for one-pass modeling (Figure 3b) to save computational costs, mapped to the actual dimensions using reflection-free boundaries. For mapping modeling (Figure 3c), it was created based on the actual dimensions of 5 mm × 50 mm × 50 mm. With a hybrid approach of explicit and implicit, the highly dynamic response behavior of materials during LSP and the final residual stress distribution were calculated sequentially.

### 3.2. Material Modeling

During LSP, shock waves cause highly dynamic responses in materials with strain rates up to 106 s^−1^. Our self-developed Ma-Wang material model accurately describes the dynamic response behaviors of metallic materials (including AA6061-T6) at ultra-high strain rates up to 108 s^−1^ [45,46,47,48] and can be applied in this study. The specific equation, written as $\sigma =\left\{\left[\sigma_{Y0}+A{\left(\varepsilon^{p}\right)}^{n}\right]+\left(\alpha \varepsilon^{p}+\beta \right)\left[1-\frac{\sigma_{Y0}+A{\left(\varepsilon^{p}\right)}^{n}}{\sigma_{cr}}\right]ln\left(\frac{{\dot{\varepsilon }}^{p}}{{\dot{\varepsilon }}_{s}^{p}}\right)+Bln\left(\frac{{\dot{\varepsilon }}^{p}}{{\dot{\varepsilon }}_{u}^{p}}\right)\right\}{\left[\frac{sigmoid\left(-\frac{T-T_{a}}{T_{m}-T_{a}}b\right)}{sigmoid\left(-\frac{T_{R}-T_{a}}{T_{m}-T_{a}}b\right)}\right]}^{m}$, represents the dependence of flow stress (σ) on strain ($\varepsilon^{p}$), strain rate (${\dot{\varepsilon }}^{p}$), and temperature (T), taking into account strain hardening, full-range strain rate hardening (10^−3^ s^−1^–109 s^−1^), and thermal softening/hardening. The model parameters applicable to AA6061-T6 are listed in Table 2, and their validity has been proven [46,48].

## 4. Results and Discussion

### 4.1. Distribution Characteristics of Laser Shock Peening-Induced Residual Stresses

**Figure 4:**
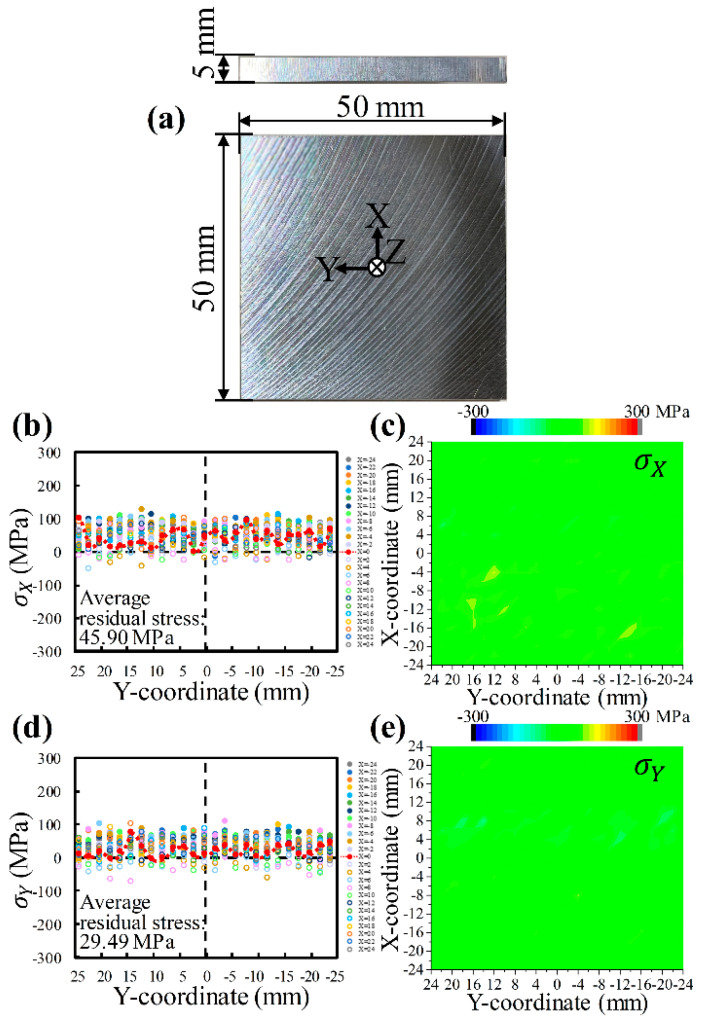
Initial residual stresses on AA6061-T6 sheet surface before laser shock peening: (**a**) photograph of an actual specimen; $\sigma_{X}$ (**b**) distribution along the Y-direction and (**c**) mapping; $\sigma_{Y}$ (**d**) distribution along the Y-direction and (**e**) mapping. *(**c**,**e**) were made using the “Contour–Color Fill” function of Origin 2024*.

Before LSP, the initial residual stresses on the AA6061-T6 sheet surface were characterized to serve as a comparison, as shown in Figure 4. The residual stresses $\sigma_{X}$ and $\sigma_{Y}$ exhibit similarities; both are predominantly tensile, ranging from 0 to 100 MPa. The error bars for the residual stresses $\sigma_{X}$ and $\sigma_{Y}$ are about ±26.98 MPa and ±28.13 MPa, respectively. The average residual stresses are 45.90 MPa (standard deviation 30.80 MPa, Figure 4b,c) and 29.49 MPa (standard deviation 29.30 MPa, Figure 4d,e), respectively. Tensile stresses are predominantly attributed to the sheet through rolling, and the difference in average residual stresses is related to the rolling direction. The rolling marks of the sheet are visible in Figure 4a.

**Figure 5:**
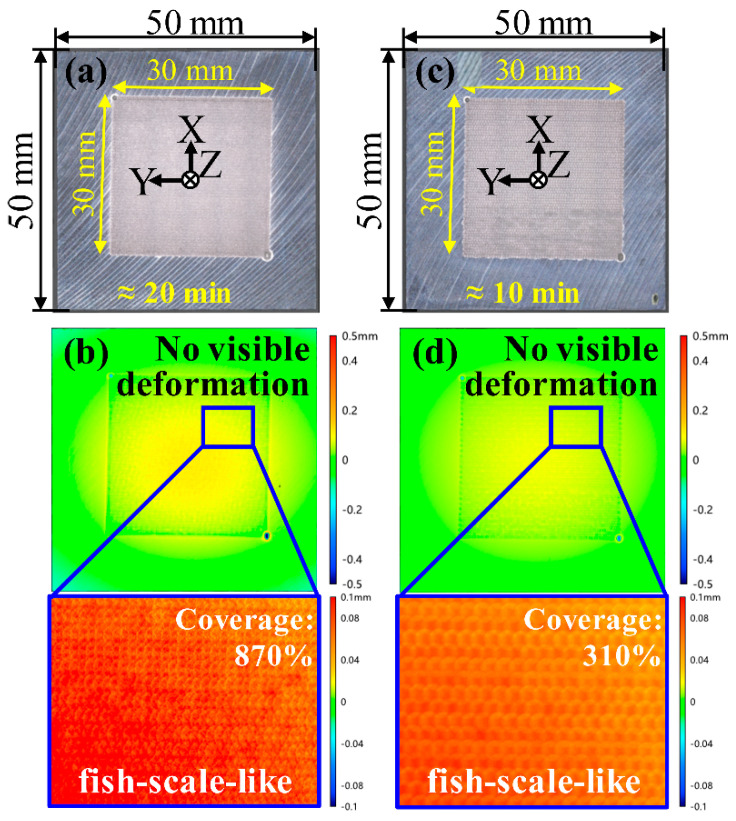
Photographs of AA6061-T6 sheets and their surface topographies after laser shock peening: (**a**,**b**) 3 mm/s scanning speed with 0.3 mm intervals; (**c**,**d**) 5 mm/s scanning speed with 0.5 mm intervals.

Figure 5 displays the AA6061 sheets after LSP at different scanning speeds and intervals, along with the corresponding surface topographies. The area of both LSP treatments is 30 mm × 30 mm. There is no visible deformation in either case, and the surface texture is fish-scale-like (as is well known), as shown in Figure 5b,d. However, the LSP processing time varies significantly. The LSP was performed at a scanning speed of 3 mm/s with intervals of 0.3 mm and took 20 min (Figure 5a). Its scanning speed of 5 mm/s with intervals of 0.5 mm takes only 10 min (Figure 5c), saving half the time. From an efficiency standpoint, faster scanning speeds and larger intervals are preferable. Notably, there is a significant difference in the LSP coverage. With the shortening of LSP processing time, the coverage dropped from 870% to 310%. Therefore, it is imperative to compare the residual stress distributions in both cases.

**Figure 6:**
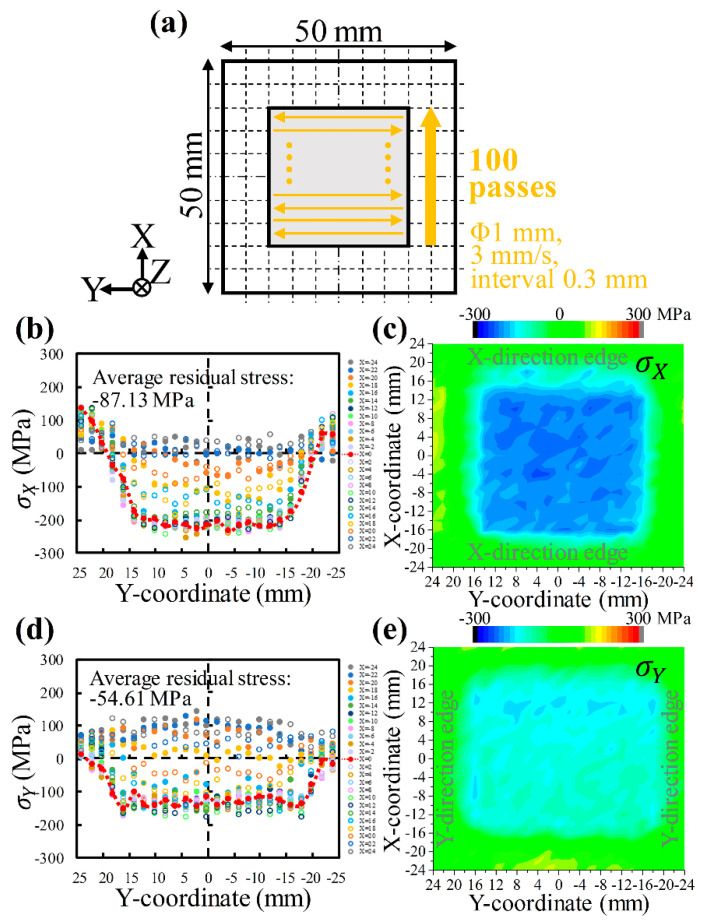
Residual stresses on AA6061-T6 sheet surface after laser shock peening: (**a**) specific scanning strategy at a scanning speed of 3 mm/s with 0.3 mm intervals; $\sigma_{X}$ (**b**) distribution along the Y-direction and (**c**) mapping; $\sigma_{Y}$ (**d**) distribution along the Y-direction and (**e**) mapping. *(**c**,**e**) were made using the “Contour–Color Fill” function of Origin 2024 software*.

Figure 6 shows the specific scanning strategy at a scanning speed of 3 mm/s with 0.3 mm intervals, as well as the corresponding distributions of residual stresses $\sigma_{X}$ and $\sigma_{Y}$ on the sheet surface. Comparing Figure 4 and Figure 6 indicates that this LSP condition effectively eliminates the initial tensile residual stresses and evokes noticeable compressive residual stresses. The residual stress $\sigma_{X}$ induced by LSP is approximately −200 MPa (Figure 6b,c), which gradually decreases or even becomes tensile residual stress away from the treatment area to achieve stress balance. The residual stress $\sigma_{Y}$ is around −150 MPa (Figure 6d,e) and decreases to zero at the Y-direction edge position. The error bars for the residual stresses $\sigma_{X}$ and $\sigma_{Y}$ are about ±18.15 MPa and ±18.46 MPa, respectively. It is well known that the normal residual stress on a free surface is zero. Due to stress balance, the residual stress $\sigma_{Y}$ appears tensile near the X-direction edge. It is worth noting that the compressive residual stress of $\sigma_{X}$ is significantly higher than that of $\sigma_{Y}$, suggesting the anisotropy of the LSP-induced residual stresses. Similar phenomena have been reported [2,38,39], but in the meantime, many researchers still consider LSP-induced residual stresses to be isotropic [37,49]. Hence, this study pays more attention to this anisotropy.

**Figure 7:**
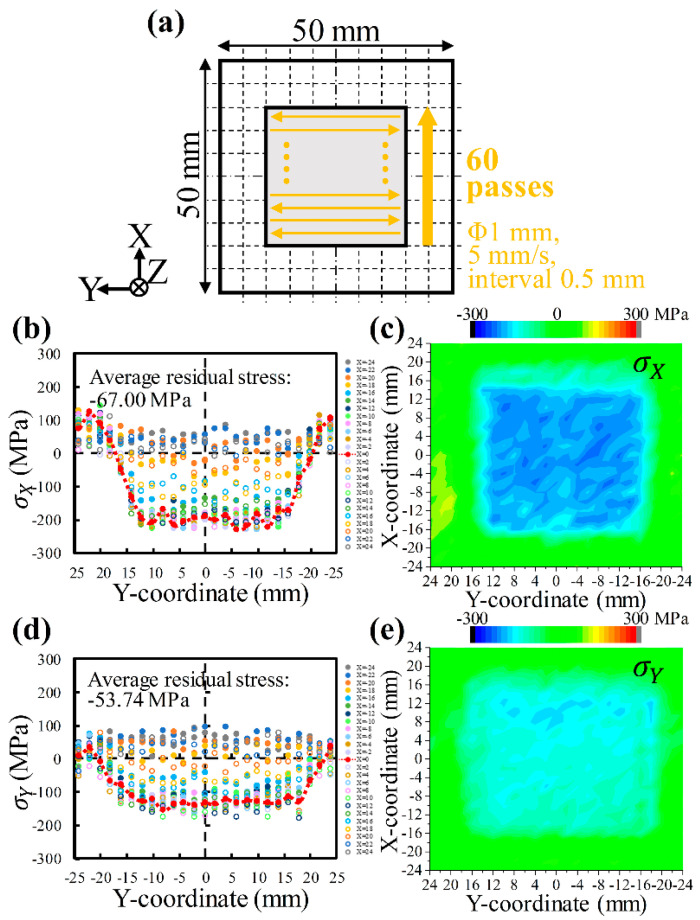
Residual stresses on AA6061-T6 sheet surface after laser shock peening: (**a**) specific scanning strategy at a scanning speed of 5 mm/s with 0.5 mm intervals; $\sigma_{X}$ (**b**) distribution along the Y-direction and (**c**) mapping; $\sigma_{Y}$ (**d**) distribution along the Y-direction and (**e**) mapping. *(**c**,**e**) were made using the “Contour–Color Fill” function of Origin 2024 software*.

The specific scanning strategy at a scanning speed of 5 mm/s with 0.5 mm intervals and the corresponding distributions of residual stresses $\sigma_{X}$ and $\sigma_{Y}$ on the sheet surface are shown in Figure 7. The residual stress distribution is consistent with that in Figure 6. Significant compressive residual stresses exist due to LSP, although they are slightly lower. The decrease in coverage leads to lower compressive residual stresses in both the X and Y directions, which can be seen more clearly by comparing Figure 6c,e with Figure 7c,e. Moreover, in this case, the LSP-induced residual stress $\sigma_{X}$ is also about −200 MPa (Figure 7b,c). The residual stress $\sigma_{Y}$ is also about −150 MPa (Figure 7d,e), which is obviously lower than the residual stress $\sigma_{X}$, further supporting the anisotropy of the LSP-induced residual stresses. The error bars for the residual stresses $\sigma_{X}$ and $\sigma_{Y}$ are about ±20.93 MPa and ±21.88 MPa, respectively. Comparing Figure 6 and Figure 7 illustrates that appropriately increasing the scanning speed and interval does not necessarily weaken the function of LSP. Both can produce compressive residual stresses. However, compared to a scanning speed of 3 mm/s with intervals of 0.3 mm, a scanning speed of 5 mm/s with intervals of 0.5 mm halves the time required to treat the area of 30 mm × 30 mm, resulting in a much more efficient surface treatment to improve the fatigue life and wear resistance of aerospace and automative components (e.g., engine blades and leaf springs). This finding provides a new insight into improving LSP efficiency. Meanwhile, it also signifies the necessity of optimizing LSP process parameters scientifically. Subsequent sections are exhaustive through numerical simulations, focusing on three typical process parameters: LSP coverage, LSP energy, and scanning path.

### 4.2. Influences of Laser Shock Peening Coverage and Energy

**Figure 8:**
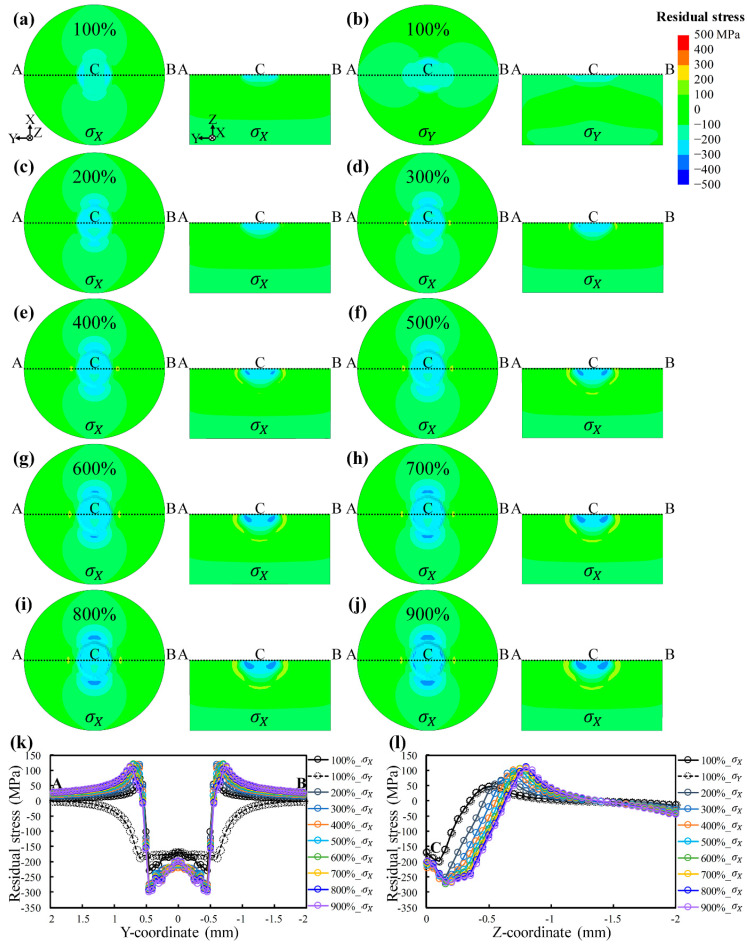
Influence of laser shock peening coverage on residual stresses of AA6061-T6 sheets: (**a**,**b**) 100%; (**c**) 200%; (**d**) 300%; (**e**) 400%; (**f**) 500%; (**g**) 600%; (**h**) 700%; (**i**) 800%; (**j**) 900%. Comparison of (**k**) residual stresses on the top surface and (**l**) residual stresses along the Z-direction with different coverages.

LSP coverage is closely related to processing efficiency, as shown in Section 4.1. Hence, Figure 8 displays the residual stress distributions at different LSP coverages to clarify the individual influence of the coverage. Figure 8a–j exhibit the residual stress distributions, and Figure 8k,l compare the residual stresses on the top surface and residual stresses along the depth direction, respectively. It is clearly shown that LSP induces compressive residual stresses on the top surface, which increase to the maximum and then decrease to zero or even transform into tensile ones along the depth direction. This is a typical distribution of residual stresses along depth after LSP [26], which is not detailed here. The residual stress distributions of $\sigma_{X}$ and $\sigma_{Y}$ for the same coverage are identical (Figure 8a,b), indicating that the coverage is not the underlying cause of residual stress anisotropy. As the LSP coverage increases, the compressive residual stresses gradually increase. Nonetheless, the increase in residual stresses is slight, except for the transition from 100% to 200%, which is more clearly visible in Figure 8k,l. In addition, the greater the coverage, the higher the residual stress, but the longer the scanning time required. Too high coverage (400%→900%) can lead to high tensile residual stresses and localized severe plastic deformation near the surface, which can adversely affect the improvement of mechanical properties. This effect is also particularly evident along the depth direction in Figure 8j. The above identifies the individual role of LSP coverage and simultaneously provides theoretical support for the choice of LSP coverage. Choosing appropriate coverage is not only beneficial to LSP efficiency but also to LSP effectiveness.

**Figure 9:**
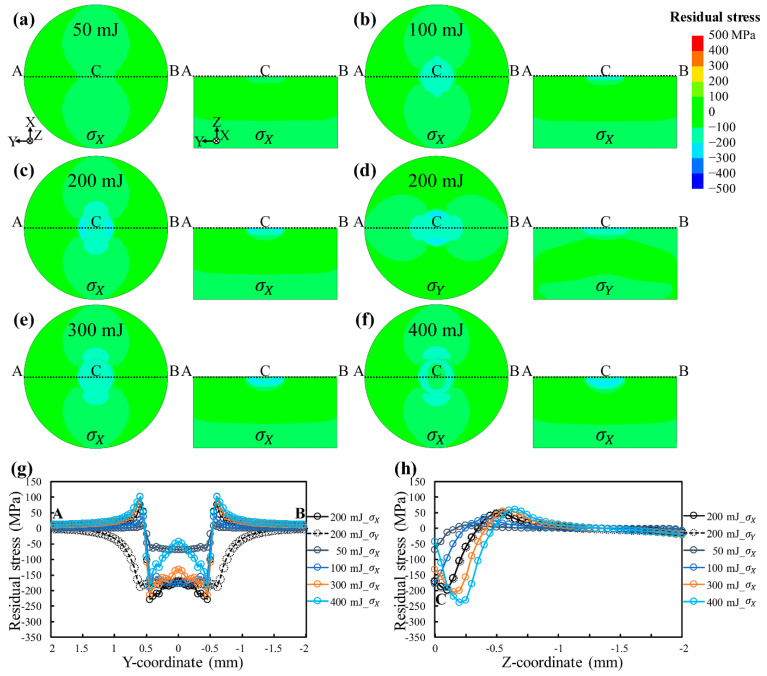
Influence of laser shock peening energy on residual stresses of AA6061-T6 sheets: (**a**) 50 mJ; (**b**) 100 mJ; (**c**,**d**) 200 mJ; (**e**) 300 mJ; (**f**) 400 mJ. Comparison of (**g**) residual stresses on the top surface and (**h**) residual stresses along the Z-direction with different energies.

LSP energy is a vital parameter correlating with laser power intensity (Section 2.1). Figure 9 shows the residual stress distributions at different LSP energies to illustrate the individual influence of the energy. Figure 9a–f present the residual stress distributions, and Figure 9g,h compare the residual stresses on the top surface and residual stresses along the depth direction, respectively. It is evident that the residual stress distributions of $\sigma_{X}$ and $\sigma_{Y}$ for the same energy are identical, which specifies that the LSP energy is also not the source of the anisotropy (Figure 9c,d). The increase in LSP energy can significantly increase the induced compressive residual stresses on the top surface and along the depth direction. Increasing energy does not change the distribution mode of residual stresses along the depth direction: compressive stress exists on the surface and first increases and then decreases along the depth direction. However, excessive energy can cause a weakening of the surface compressive residual stresses, especially at the center of the LSP-treated point, as can be seen more clearly in Figure 9g. Also, too much energy can cause an overall large plastic deformation of the sheet, the so-called laser peen forming. Consequently, selecting the appropriate LSP energy is essential to guaranteeing the quality of LSP-treated components. The above identifies the individual role of LSP energy while offering theoretical support for its selection.

### 4.3. Residual Stress Dependence on Scanning Path

#### 4.3.1. Singe-Pass Scanning

**Figure 10:**
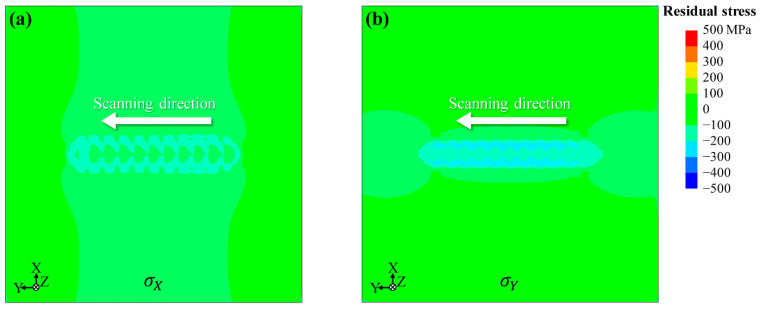
Comparison of (**a**) $\sigma_{X}$ and (**b**) $\sigma_{Y}$ residual stress distributions for one-pass scanning at a scanning speed of 5 mm/s with 0.5 mm intervals.

The experimental results show that the LSP-induced residual stress distribution exhibits typical anisotropic characteristics, with residual stress $\sigma_{X}$ being higher than residual stress $\sigma_{Y}$. This feature in this study remains consistent regardless of LSP coverage and energy, as demonstrated in Section 4.2. Presumably, it is related to the scanning path. To simplify the issue, one-pass LSP simulation at a scanning speed of 5 mm/s with 0.5 mm intervals was carried out, and the results are shown in Figure 10. It is the anisotropic nature of the residual stress distribution. The higher residual stress in the scanning direction can be explained as follows: The superposition of multiple LSPs results in a larger plastic deformation region in the scanning direction, which increases the corresponding compressive residual stress, i.e., residual stress $\sigma_{Y}$. This suggests that the anisotropy of the induced compressive residual stresses depends on the scanning path. In other words, the scanning path is the key to causing the anisotropy.

#### 4.3.2. Mapping Scanning

**Figure 11:**
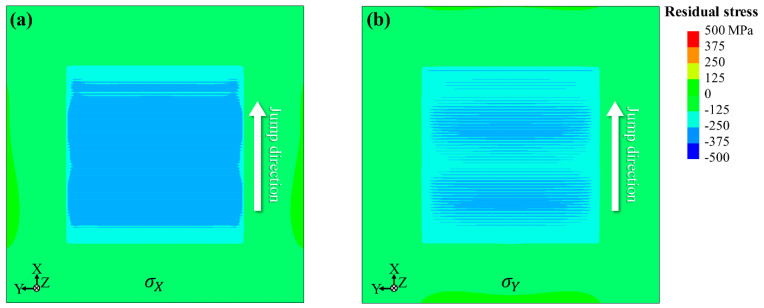
Comparison of (**a**) $\sigma_{X}$ and (**b**) $\sigma_{Y}$ residual stress distributions for mapping scanning at a scanning speed of 5 mm/s with 0.5 mm intervals.

To further comprehend this anisotropy, a mapping LSP simulation was performed at a scanning speed of 5 mm/s with 0.5 mm intervals, and the results are shown in Figure 11. Overall, the residual stress $\sigma_{X}$ is noticeably higher than the residual stress $\sigma_{Y}$. These simulated distribution characteristics agree well with the experimental measurements and support the validity of the model. Although Figure 10 exhibits higher residual stress $\sigma_{Y}$ in one-pass LSP, the residual stress $\sigma_{X}$ is higher in mapping LSP. This disparity is reasonable because, in mapping LSP, there is a jump direction in addition to the scanning direction. The implementation of subsequent LSP passes causes the overlapping of residual stresses in the jump direction, thereby increasing the residual stress $\sigma_{X}$. It further supports the idea that the scanning path is the key to causing the anisotropy. The above reproduces the anisotropy of LSP-induced residual stresses, identifying the key factor contributing to anisotropy. On the other hand, it provides theoretical support for optimizing the scanning path to aid mechanical properties. The current study focuses on the residual stresses induced by LSP, and in the future, we will pay more attention to the fatigue performance improved by LSP-induced residual stresses.

## 5. Conclusions

LSP experiments were carried out on AA6061-T6 sheets, and residual stresses were measured by the XRD (cosα) method. A series of one-point, one-pass, and mapping LSP simulation models were established to elucidate the formation mechanism of LSP residual stresses and the dependent factors influencing the distribution characteristics. Most current LSP simulations focus on residual stresses caused by one or several laser pulses. Moreover, this study identified the scanning path as the key to causing the anisotropy. It provides theoretical support for quickly and effectively tailoring LSP residual stresses, which has great economic value for aerospace and automotive fields. Furthermore, it is possible to design scanning paths to minimize failures in different directions. In the future, we will further explore different alloys and more complex geometries. The conclusions are as follows:

(1) Measurement results indicate that LSP enables notable compressive residual stresses on specimen surfaces without visible deformations. Nevertheless, the induced compressive residual stresses are anisotropic. The zigzag scanning path gives the LSP-treated surface a fish-scale-like appearance.
(2) The individual influences of LSP coverage and energy are illustrated by modeling. Both increases contribute to the induced compressive residual stresses. However, the greater the coverage, the longer it takes to scan. Excessive LSP energy reduces the surface compressive residual stress. Choosing appropriate process parameters is critical to balancing LSP efficiency and effectiveness.
(3) LSP-induced residual stress anisotropy is closely related to the scanning path. Residual stresses are significantly higher in the scanning direction for one-pass LSP and in the jump direction for mapping LSP. The main reason is that the residual stresses are overlapped by subsequent LSP processing. It identifies the key role of the scanning path for residual stress anisotropy.

## Figures and Tables

**Table 1:** Specifications of the nanosecond laser used.

Wavelength	Pulse Duration	Pulse Frequency	Pulse Energy
532 nm	8 ns	10 Hz	~500 mJ

**Table 2:** Model parameters for describing the response behavior of AA6061-T6 in LSP simulations.

Basic Properties	Symbols	AA6061-T6
Young’s modulus	E	68.9 GPa
Poisson’s ratio	v	0.33
Density	D	2703 kg·m^−3^
Specific heat	c	875 J·kg^−1^·°C^−1^
Fraction of plastic work converted into heat	η	0.9
(*Ma-Wang material model*)		
Strain hardening	$\sigma_{Y0}$	324 MPa
	A	114 MPa
	n	0.42
Full-range strain rate hardening	${\dot{\varepsilon }}_{s}^{p}$	0.001 s^−1^
	α	4.52 MPa
	β	0.65 MPa
	$\sigma_{cr}$	1.5 GPa
	${\dot{\varepsilon }}_{u}^{p}$	200 s^−1^
	B	22 MPa
Thermal softening/hardening	$T_{R}$	25 °C
	$T_{m}$	652 °C
	$T_{a}$	186.7 °C
	b	10.00
	m	0.05

## Data Availability

The original contributions presented in the study are included in the article, further inquiries can be directed to the corresponding authors.
